# Quantitative Whole Body Biodistribution of Fluorescent-Labeled Agents by Non-Invasive Tomographic Imaging

**DOI:** 10.1371/journal.pone.0020594

**Published:** 2011-06-22

**Authors:** Kristine O. Vasquez, Chelsea Casavant, Jeffrey D. Peterson

**Affiliations:** Department of Applied Biology, PerkinElmer, Inc., Boston, Massachusetts, United States of America; Genentech, United States of America

## Abstract

When small molecules or proteins are injected into live animals, their physical and chemical properties will significantly affect pharmacokinetics, tissue penetration, and the ultimate routes of metabolism and clearance. Fluorescence molecular tomography (FMT) offers the ability to non-invasively image and quantify temporal changes in fluorescence throughout the major organ systems of living animals, in a manner analogous to traditional approaches with radiolabeled agents. This approach is best used with biotherapeutics (therapeutic antibodies, or other large proteins) or large-scaffold drug-delivery vectors, that are minimally affected by low-level fluorophore conjugation. Application to small molecule drugs should take into account the significant impact of fluorophore labeling on size and physicochemical properties, however, the presents studies show that this technique is readily applied to small molecule agents developed for far-red (FR) or near infrared (NIR) imaging. Quantification by non-invasive FMT correlated well with both fluorescence from tissue homogenates as well as with planar (2D) fluorescence reflectance imaging of excised intact organs (r^2^ = 0.996 and 0.969, respectively). Dynamic FMT imaging (multiple times from 0 to 24 h) performed in live mice after the injection of four different FR/NIR-labeled agents, including immunoglobulin, 20–50 nm nanoparticles, a large vascular imaging agent, and a small molecule integrin antagonist, showed clear differences in the percentage of injected dose per gram of tissue (%ID/g) in liver, kidney, and bladder signal. Nanoparticles and IgG1 favored liver over kidney signal, the small molecule integrin-binding agent favored rapid kidney and bladder clearance, and the vascular agent, showed both liver and kidney clearance. Further assessment of the volume of distribution of these agents by fluorescent volume added information regarding their biodistribution and highlighted the relatively poor extravasation into tissue by IgG1. These studies demonstrate the ability of quantitative FMT imaging of FR/NIR agents to non-invasively visualize and quantify the biodistribution of different agents over time.

## Introduction

Pharmacokinetic, biodistribution, and metabolic clearance characteristics are important properties that can influence the overall efficacy of novel therapeutics, imaging agents, and macromolecular delivery vectors [1], [2], [3], [4], [5]. In order to achieve an effective level of agent in the target tissues (*i.e.* sites of disease, inflammation, tissue remodeling, tumor growth, or other biological changes) the agent should extravasate from circulating blood to the tissue of interest or site of action, either accumulating non-specifically, binding to a molecular target, and/or undergoing cellular internalization. The type of agent, dictated by physicochemical properties such as size, charge, and chemical composition, will not only influence pharmacokinetics and biodistribution but will also influence the volume of distribution, the route of metabolism, and clearance. In the development of both therapeutic agents and imaging agents, all of these parameters contribute to the risk assessment of an agent with regard to efficiency of targeting, assessment of off-target accumulation, and prediction of potential sites of adverse reactions.

Preclinical biodistribution and pharmacokinetics data for investigational agents are routinely obtained in animal studies using radiolabeled materials [6]. Many of these studies employ post-mortem scintillation counting of the labeled radioactivity in excised organs and tissues [7], [8]. Additional understanding of the kinetic changes in biodistribution has generally required either the sacrifice of multiple animals at multiple time points or the use of non-invasive nuclear imaging techniques. PET and SPECT imaging can provide 3-dimensional spatial distribution datasets of radio-labeled imaging agents or therapeutics. By combining these imaging approaches with CT, which provides additional anatomical context, the quantitative accuracy of the PET and SPECT data can be improved [9]. However, widespread use of PET or SPECT for biodistribution studies can be limited by cost, the restricted availability of the radionuclide-labeled agents, and the extra precautions and safety guidelines required for working with radioactivity. As an alternative, some researchers have examined near infrared fluorescent imaging as a convenient, easy, and rapid means of assessing agent biodistribution in excised organ tissues, again requiring the sacrifice of experimental animals [7], [10], [11]. In support of that early work, we present the FMT as a robust and sensitive instrument enabling detection, visualization, and quantification of fluorescence distributed throughout the body of living mice [12], [13], [14], [15], [16], [17], [18], [19] in a manner analogous to PET imaging. The natural extension of this FMT technology to full-body biodistribution measurements may offer an approach to augment whole body PET and SPECT approaches. An additional benefit of the use of fluorescence for biodistribution studies is the unique ability to simultaneously distinguish multiple agents after IV injection. Spectrally resolved optical imaging offers the opportunity to track up to four separate agents, each labeled with a different fluorophore that emits at a different wavelength. Upon simultaneous injection of two labeled agents, the relative biodistributions of two labeled agents could be directly and quantitatively compared.

In these studies, we built upon recent advances in optical tomographic imaging and FR/NIR agents to demonstrate the utility of non-invasive FMT to detect, localize, and quantify agent biodistribution in the major organ systems. For this, we used empirical testing with a variety of FR/NIR-labeled agents to establish 3D regions of interest (ROI) that optimally capture different organs with minimal overlap. BSA-VT680XL FMT results agreed well with comprehensive *ex vivo* assessments of organs, both by 2D organ imaging as well as by quantification of fluorescence within tissue homogenates. Subsequent dynamic FMT imaging (multiple time points from 0 to 24 h) was performed using four different types of FR/NIR-labeled imaging agents, including a 1.4 kDa drug-like integrin antagonist [20], a 250,000 kDa vascular agent [21], [22], 20–50 nm fluorescent nanoparticles [23], and fluorophore-labeled proteins (BSA and IgG1). Careful, quantitative interpretation of temporal imaging datasets, taking into account the signal contributed by circulating agent, yielded useful information regarding the amount and time course of agent extravasation and localization into specific tissues. These imaging agents showed differences in liver, kidney, and bladder accumulation, with the nanoparticles and IgG1 favoring liver/kidney signal, the small molecule agent showing expected rapid kidney and bladder clearance, and the high molecular weight vascular agent showing both liver and kidney clearance. Additional assessment of the fluorescence volumes quantified within the animals provided a simple indication of the volume of distribution differences, with the integrin agent showing much greater dissemination into tissues than larger agents, despite its very short blood half-life, and immunoglobulin showing expected poor extravasation into tissue.

FMT biodistribution assessment, by virtue of its safety and ease of use, may be ideal for the routine study of small molecule imaging agents or larger therapeutic biomolecules (antibodies or proteins minimally affected by fluorophore labeling). Future combination of FMT with CT imaging or fluorescent contrast agents, to provide anatomical co-registration, may further advance the robustness of biodistribution imaging.

## Results

### Control studies to establish tomographic imaging analysis regions for different organ systems

**Figure 1:**
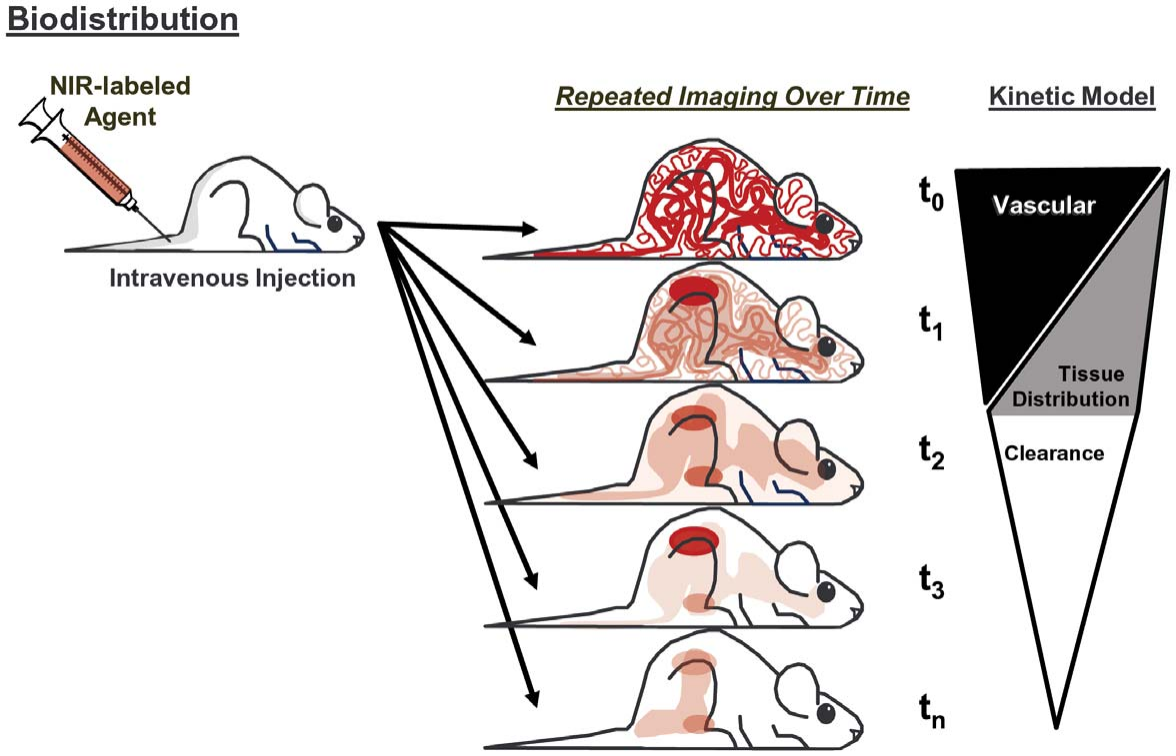
Diagrammatic representation of the FMT biodistribution model. Mice are injected with NIR-labeled agents at time 0 and imaged by FMT immediately, at the time when all of the signal would be attributed to the vasculature. At regular times thereafter, mice are reimaged to assess the dynamic changes in tissue distribution, including kinetics of extravasation into specific organ regions as well as the kinetics of tissue clearance.

**Figure 2:**
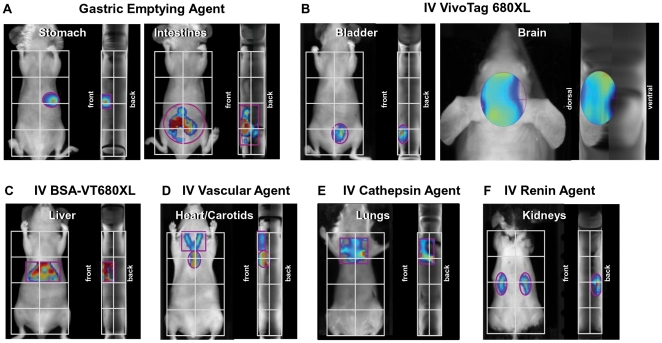
Establishing FMT image analysis techniques for accurate assessment of specific organ systems. Nude or BALB/c mice received administration of a variety of NIR fluorescence imaging agents to help to define specific organ regions and the appropriate placement of regions of interest, ROI, for quantification. **A**, GastroSense 680 was orally gavaged, and mice were imaged immediately to visualize the stomach and to place a 3-dimensional ROI (see front and side views). At 2 h post-gavage, the agent emptied from the stomach and moved into the intestines, allowing clear 3D intestinal ROI placement. **B**, Free fluorophore (VivoTag 680XL) was injected IV, revealing very rapid appearance in the bladder (left panels) and circulation through the vessels of the brain (right panels). **C**, BSA, known as a protein that predominantly localizes to the liver, was labeled with a NIR fluorophore (VivoTag 680XL) and injected IV to define the liver region. **D**, The large NIR vascular agent, AngioSense 680, was injected IV to provide signal defining the heart and carotid/jugular veins. **E**, Asthma-like inflammation was induced by immunization of BALB/c mice as previously described [16], and ProSense 680 was used to image the pulmonary eosinophilia cathepsin activity, thus defining the lung regions in 3D. **F**, IV ReninSense 680 was activated by normal kidney renin activity, providing signal defining the kidney regions. The resulting 3D ROIs for each of these organ/tissue regions defined the regions to be analyzed in subsequent whole body biodistribution studies, with a 2X4 grid providing a reference for proper ROI placement. Fluorescence outside of each tissue region’s ROI is omitted for clarity.

To quantitate fluorescence biodistribution in the different organs of living mice, and changes over time, studies were designed as depicted in Figure 1 using intravenous injection of fluorescent agents. For initial optimization of biodistribution analysis procedures, however, normal nude, or depilated conventional mouse strains, received injections of specific imaging agents by selected routes in order to define specific organ systems. Tomographic imaging provided 3-dimensional fluorescence datasets to accompany the photographic image acquired of each mouse’s body. A 2×4 grid was overlaid on the mouse image (from neck to tailbase) as a consistent frame of reference for analysis. A gastric emptying imaging agent, administered orally, was used to define the stomach region upon immediate tomographic imaging (left panel), and this agent emptied into the intestines (right panel) within 3 hours (Fig. 2A). The region of interest (ROI) for the stomach was placed just above and to the right of the mouse midline/centerline intersection and captured an area in the ventral third of the mouse, whereas the intestinal region was captured by a large cylindrical ROI in the abdominal region as depicted. Intravenous free fluorophore (VivoTag680XL) defined both the bladder and the brain region, specifically signal associated with blood vessels within the brain, when imaged within 15 minutes after injection (Fig. 2B). VivoTag680XL-labeled bovine serum albumin (BSA-VT680XL) accumulated in the liver at 24 h after IV injection, allowing the identification of the majority of the liver region (Fig. 2C). The ROI was purposely designed to capture approximately 70% of the liver to avoid overlap with the gastrointestinal tract and lung regions. Injection of a vascular agent, imaged immediately after injection, highlighted the heart and carotid/jugular vessels (Fig. 2D). A lysosomal cathepsin-activatable agent [16] detected eosinophil-related cathepsin activity in the lungs of asthmatic BALB/c mice after IV injection, helping to define the major regions of the lungs (Fig. 2E). The intravenous renin imaging agent was activated by normal levels of renin activity in the kidneys of C57BL/6 mice (Fig. 2F).

**Figure 3:**
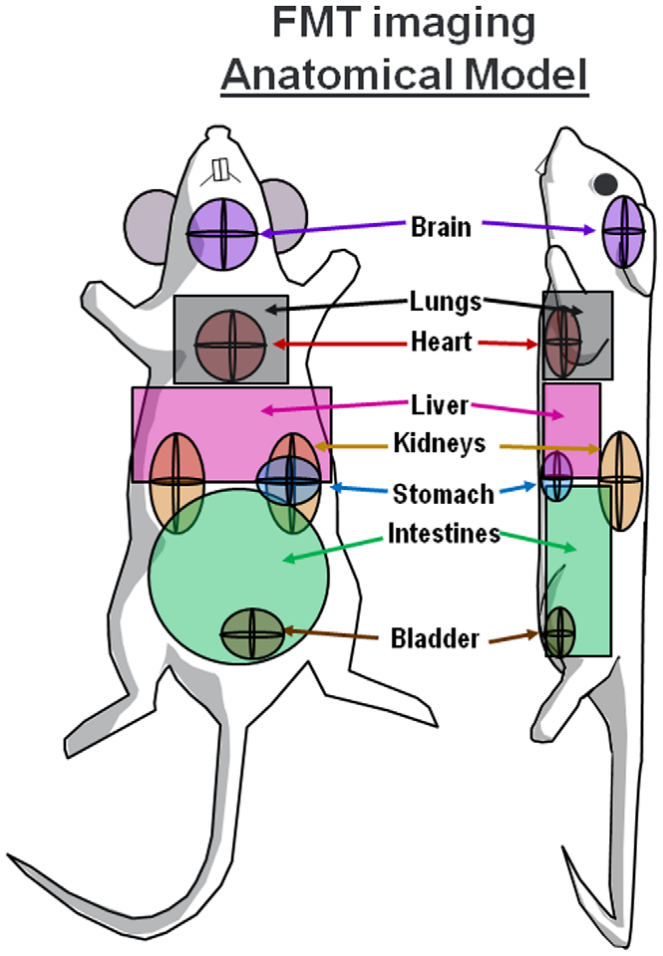
Organ region model for biodistribution study analysis. The ROIs defined in Fig. 2 using a variety of imaging agents helped to generate an analysis model that was applied to all biodistribution studies. Specific ROIs were designed to optimally capture most of each organ region while minimally capturing nearby organs. In the case of unavoidable overlap, i.e. heart/lung, bladder/intestines, and stomach/liver, careful calculations were made to subtract overlapping signal.

In the case of stomach, bladder, kidney, and heart imaging, the ROIs placed to define these organs were deliberately sized larger than the actual organs to provide sufficient room for mouse-to-mouse variability. Liver, intestine, and lung ROIs were sized as a compromise to achieve maximal specific organ capture and minimize overlap with other organ systems. The composite ROI model for FMT imaging of organ tissue biodistribution is represented in Figure 3. ROI placement for skin fluorescence assessment (not shown) was performed by placing a cylinder ROI consistently on the upper right torso to capture the outer voxel layer of fluorescence, with the grams of tissue calculated based on the volume of the ROI.

### Validating terminal *in vivo* distribution patterns of labeled bovine serum albumin (BSA) by FMT and *ex vivo* FRI tissue imaging

**Figure 4:**
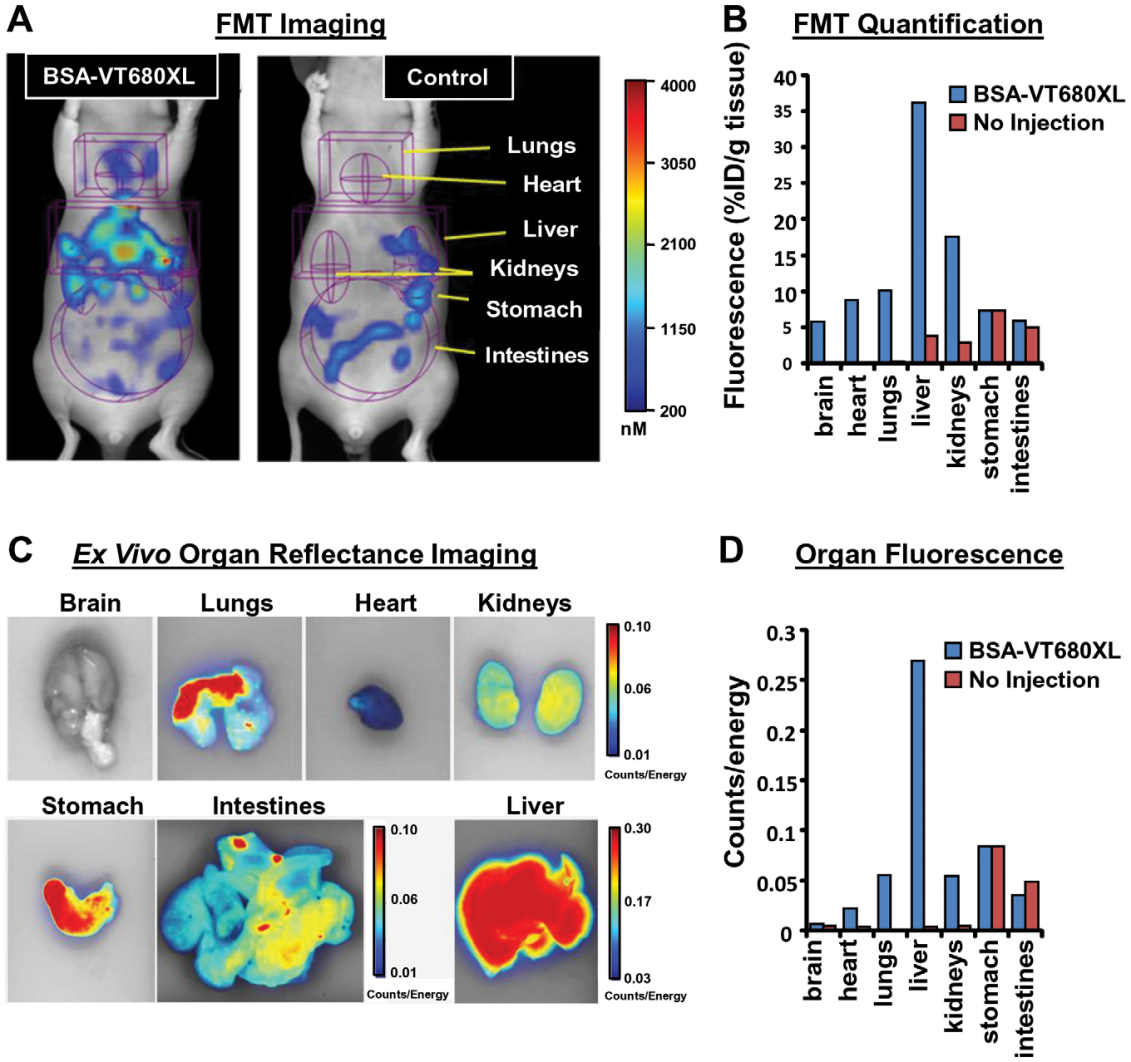
Validation of FMT biodistribution imaging techniques using NIR-labeled BSA. Nu/nu mice (n = 3 per group) received either BSA-VT680XL or no injection (Control), and mice were noninvasively imaged by FMT2500 at 24 h post-injection, a time when the agent would no longer be in circulation but would be at maximal levels in the tissue. Organ ROIs defined in Figs. 2 and 3 were applied for proper quantification of fluorescence, and the data was normalized to % injected dose per gram of tissue (assessed at study termination). Organ weights were adjusted appropriately for liver and lungs to account for incomplete capture of the full organs, based on prior studies (not shown). **A**, Tomographic images of injected and control mice revealing BSA-VT680XL distribution (left panel) and normal chow-related fluorescence in the controls (right panel). Brain fluorescence was collected in separate FMT image acquisitions (not shown). **B**, Quantification of fluorescence in injected and control mice, revealing predominant liver distribution of labeled BSA, with control mice data suggesting that all of the stomach and intestine signal could be attributed to chow-related fluorescence. **C**, Collection of specific organs was performed as an independent verification of the fluorescence quantified non-invasively by FMT. **D**, Fluorescence intensity patterns seen for the different organs, although not objectively quantitative due to the 2D acquisition, were similar to those seen by FMT imaging.

Three mice per group were administered an IV injection of 2 nmol of BSA-VT680XL and imaged non-invasively by FMT2500 at 24 h to reconstruct 3-dimensional fluorescence images (Fig. 4A, left panel). Whole-body animal imaging and image reconstruction were quick, approximately 12–15 minutes, respectively. As IV albumin would be expected to accumulate predominantly in the liver and lungs, this provided a simple approach to establishing a validated imaging system. As a control for normal chow-related fluorescence, untreated mice with no agent injection were also imaged (Fig. 4A, right panel). Organ specific ROIs were established to capture 3-dimensional anatomical regions corresponding to the heart, lungs, liver kidneys, stomach, and intestines. A separate non-invasive imaging scan of the head (not shown) captured brain fluorescence signal. Quantitative analysis of each of these regions were adjusted by taking into account the extent of any ROI overlaps. For example, the heart signal was subtracted from the lung region, part of the stomach signal was subtracted from the liver region, etc. FMT quantification, represented as the % of the injected dose per gram of tissue (%ID/g) was determined (Fig. 4B) using weights of organs collected at study termination. Appropriate adjustments were made to liver and lung weights to account for the ROI partial capture of these tissues. The liver showed the highest intensity of fluorescence per gram of tissue, followed by kidneys, lungs, heart, and brain. All of the detected stomach and intestine signal could be attributed to chow-related fluorescence, based upon similarity to the signal seen in non-injected control mice.

**Figure 5:**
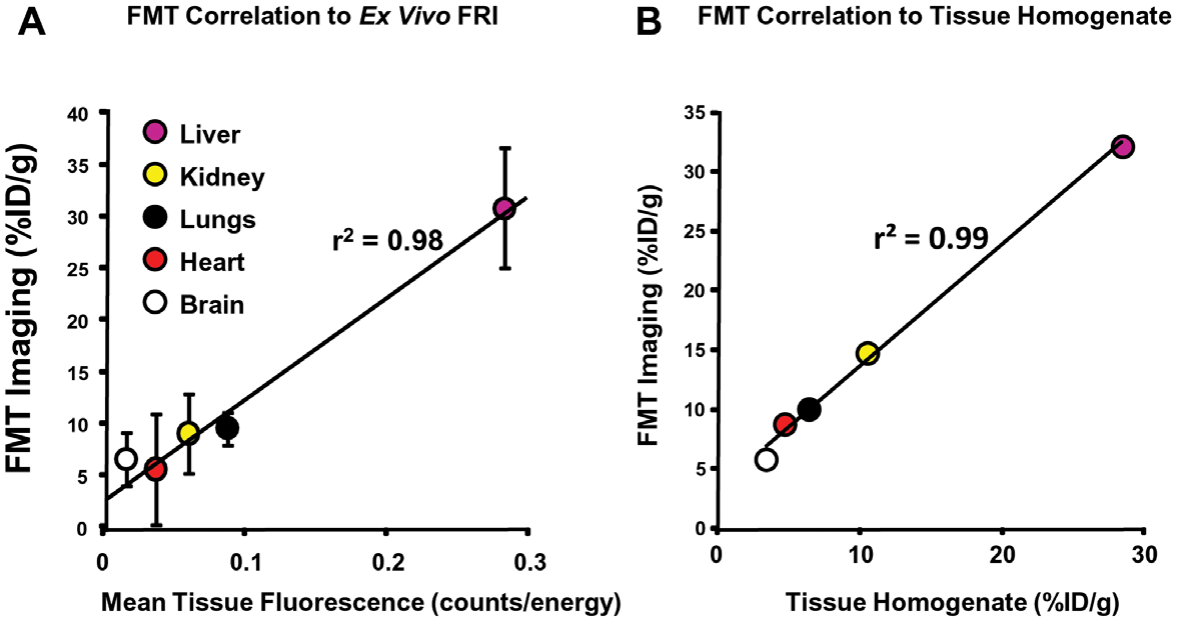
Non-invasive FMT correlation to post-mortem tissue fluorescence. Quantitative FMT imaging data from three mice injected with BSA-VT680XL was compared to post-mortem tissue assessment of organ fluorescence. **A**, Comparison of FMT quantitation to FRI gross tissue imaging results, represented in mean intensity units of counts/energy, was performed. **B**, As a second approach to validation, an independent comparison to quantitative fluorescence was performed from homogenized and diluted tissue samples, assayed in comparison to a VivoTag680XL standard curve. For this second approach, the %ID/g was calculated from quantified fluorescence and weighed organ tissue. Both comparisons yielded excellent correlation to FMT imaging, and tissue homogenate data also generated good agreement between absolute values of %ID/g.

Fluorescent-labeled BSA FMT results agreed well with comprehensive *ex vivo* assessments of organs, both by FRI organ imaging as well as by quantification of fluorescence within tissue homogenates. FRI images of the post-mortem tissues (Fig. 4C) clearly show dominant liver signal, with some signal seen in lungs, kidneys, and heart. The level of fluorescence in the stomach and intestines for the injected mice was similar to that seen in control mice (data not shown), and the mean organ signal intensities shown in Figure 3D agree well with the fluorescent signal quantified by FMT in living mice. Figure 5A further shows that FMT results correlate well with FRI, although it is no surprise that the consensus of multiple studies suggests that FRI tissue imaging underestimates signal in thicker or denser tissues such as brain, heart, and kidneys. Organs from a representative mouse were also homogenized and assayed for fluorescence levels by serial dilution, yielding a very high correlation and excellent linearity to non-invasive FMT imaging (Fig. 5B). These results also show not only that the results correlate but that the FMT %ID/g values are also in very close agreement to the independent *ex vivo* homogenate measurements.

### Assessing temporal *in vivo* whole body tissue distribution of a variety of different Fluorescent-labeled agents

**Figure 6:**
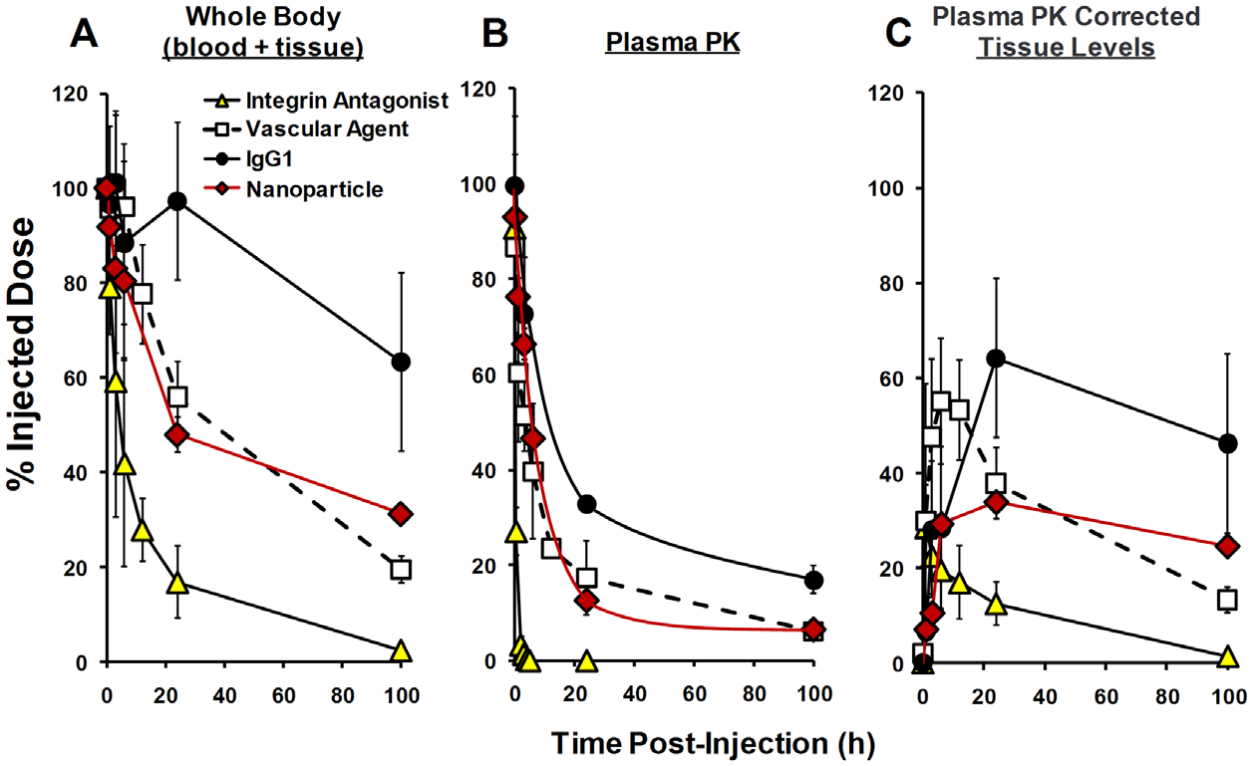
Comparison of the kinetics of whole body fluorescence changes for four different NIR-labeled agents. Four different agents were injected IV into nu/nu mice (n = 3 mice/group), and animals were imaged immediately and at 1, 3, 6, 12, 24, and 100h post injection by FMT2500. **A**, Whole body fluorescence was quantified at each time point using ROIs to capture the torso and head. Data was normalized as the percentage of the injected dose. **B**, Plasma pharmacokinetics data was collected in separate sets of animals (as described in the Materials and Methods) to provide data regarding circulating agent kinetics. Data was normalized as the percentage of the injected dose. **C**, An approximation of the kinetics of agent accumulation in tissue was established by subtracting the normalized plasma pharmacokinetic profiles from the whole body fluorescence profiles, making the assumption for each agent that the earliest imaging time point represented 100% of the agent in circulation. Corrected results reveal differences in extravasation and whole-body tissue clearance rates for all four agents.

Dynamic FMT imaging (multiple time points from 0 to 24 h) was performed using four different FR- or NIR-labeled agents, including IgG1 (150 kDa), fluorescent nanoparticles (20–50 nm diameter), a vascular imaging agent (250 kDa), and an integrin antagonist (1.4 kDa). These agents differ widely in molecular weight and chemical composition and would be expected also to differ in pharmacokinetics, tissue distribution, tissue clearance kinetics, and routes of metabolism. Careful, quantitative interpretation of temporal imaging datasets, taking into account the signal contributed by circulating agent, is an important consideration when determining the amount and time course of agent extravasation and accumulation in specific organ tissues, as illustrated in Figure 1. Whole body imaging of living mice receiving these agents (Fig. 6A) showed the changes over time in total signal (*i.e.* signal from both tissue and blood), showing three patterns of dynamic changes over time for the four agents. Little information could be obtained from this data alone with regard to the kinetics of extravasation of these agents into the tissue. However, comparison to the typical plasma pharmacokinetic profiles for these agents (Fig. 6B), normalized to %ID (and then corrected for initial loss of agent to the bladder), shows that the rate of decline of signal in the plasma was much more rapid than seen in the whole body imaging, suggesting that each of these agents was distributing to the tissue to some degree. By imaging immediately after agent injection, we were able to normalize the starting time point FMT quantification and the plasma levels. By subtracting the normalized plasma PK data from the whole body imaging results (Fig. 6C), quantification of the tissue distribution dynamic profile was achieved. Each of the four agents used in this study showed distinct profiles of tissue distribution. Most notably, the small molecule integrin imaging agent cleared rapidly from the blood and achieved ∼30% of the injected dose in the tissue at 1 h. In the absence of high plasma levels to drive tissue accumulation, this agent started clearing from the tissue immediately thereafter with a tissue half-life of approximately 20 h. The vascular and nanoparticle agents, despite sharing very similar plasma pharmacokinetics, differed in their tissue distribution kinetics, with the vascular agent showing peak tissue signal (∼60%ID) at 5 h and a tissue half-life of ∼40 h and the nanoparticles showing substantially lower peak tissue accumulation (∼35%ID) but with a very long residence time (tissue t_1/2_>100 h). IgG1 showed the slowest extravasation into tissue (peaks at 24 h) and also has a tissue half-life >100 h.

### Assessing dynamic *in vivo* organ distribution patterns of a variety of different fluorescent-labeled agents

**Figure 7:**
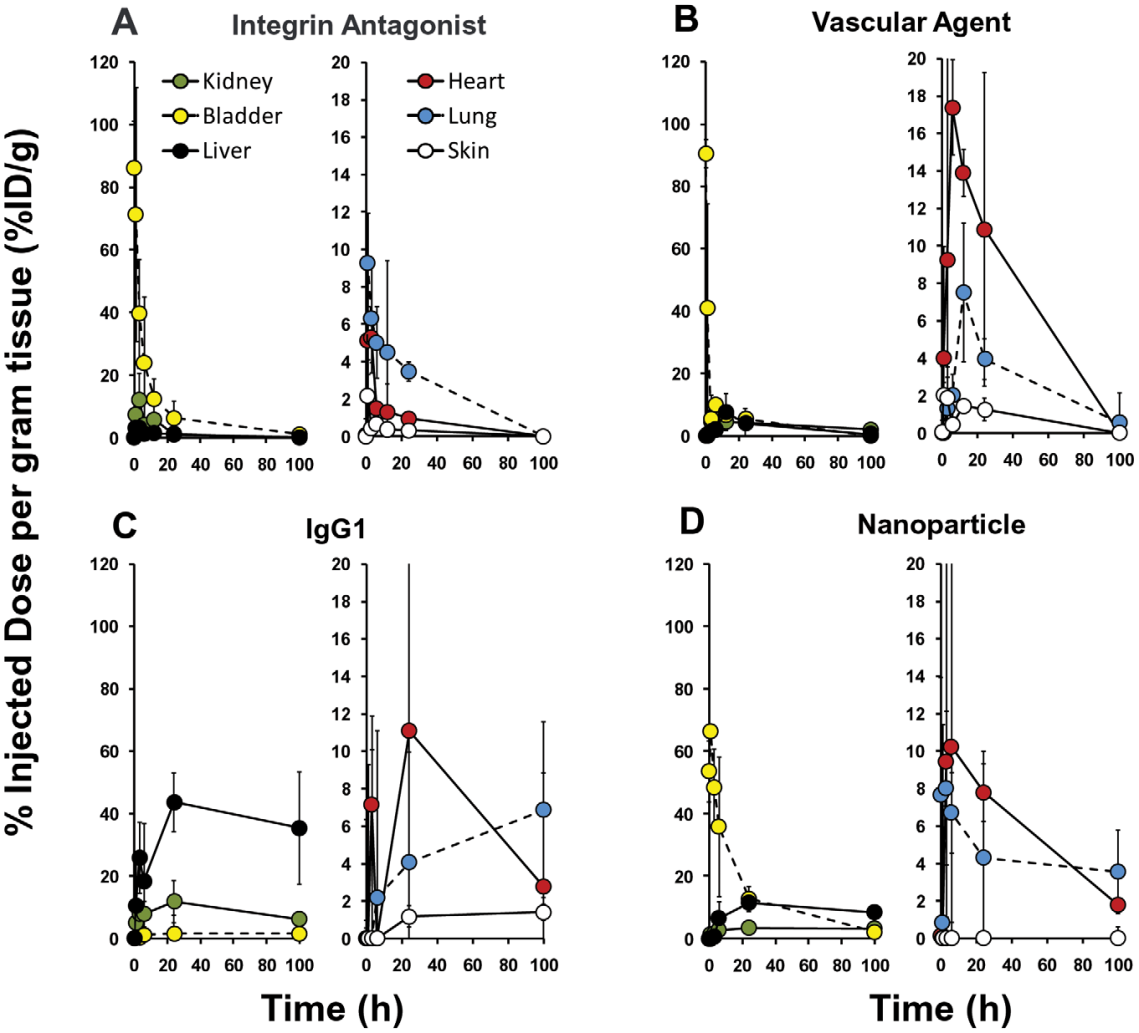
Comparison of the kinetics of organ biodistribution for four different NIR-labeled agents. Mice from the study in Fig. 6 were further assessed for kinetic changes in fluorescence patterns in the different organ systems using techniques described in Figs 2 and 3. The plasma level adjusted quantification of the kinetics of agent accumulation in each organ region was established by subtracting the normalized plasma pharmacokinetic profiles from each organ’s fluorescence profile, making the assumption for each agent that the earliest imaging time point represented 100% of the agent in circulation within that organ. Corrected results for **A**, the integrin antagonist, **B**, the vascular agent, **C**, IgG1, and **D**, nanoparticles, reveal differences in the kinetics of extravasation into each organ and kinetic clearance patterns for all four agents.

Further analysis of whole body imaging datasets for the four agents represented in Figure 6 yielded temporal information on liver, lung, kidney, heart, bladder, and skin signal (Fig. 7). Again, the imaging data acquired by FMT captured signal originating from both the organ tissue and the blood within the vessels of the organs. This assessment alone was somewhat misleading, particularly when examining very early time points at which agent levels were high in the circulation. A similar approach to the whole body tissue assessment shown in Figure 6 (*i.e.* subtraction of normalized PK curves from imaging data) was applied to each organ system, again making the assumption that at time zero all of the signal can be attributed to the blood. The plasma PK values were normalized for each organ and subtracted from each organ’s total %ID/g value (essentially “zeroing” each organ for the initial imaging time point). The resulting plasma corrected tissue levels gave a clear picture of the kinetics of extravasation of each agent into each organ tissue. Bladder signal was not corrected because we have found minimal vascular contribution to bladder fluorescence.

The integrin antagonist showed very early bladder signal due to its low molecular weight and rapid kidney clearance (Fig. 7A), with very low signal (*i.e.* less than 5–6%ID/g) seen in other tissues. The vascular and nanoparticle agents (Fig. 7B&D) also show a very early bladder signal spike upon imaging, attributed to a small percentage of trapped free fluorophore that shows as 10–12%ID at the starting time point with rapid disappearance. The overall profiles of these two agents in other tissues were similar, but they showed differences in liver and heart signal. In contrast, IgG1 signal was dominated by liver accumulation as well as long term general accumulation in most tissues (Fig. 7C) that was highly variable. There was negligible signal in the bladder region, revealing that bladder vasculature generally contributes only minimally to the overall signal in the bladder, even at peak levels in circulation.

**Figure 8:**
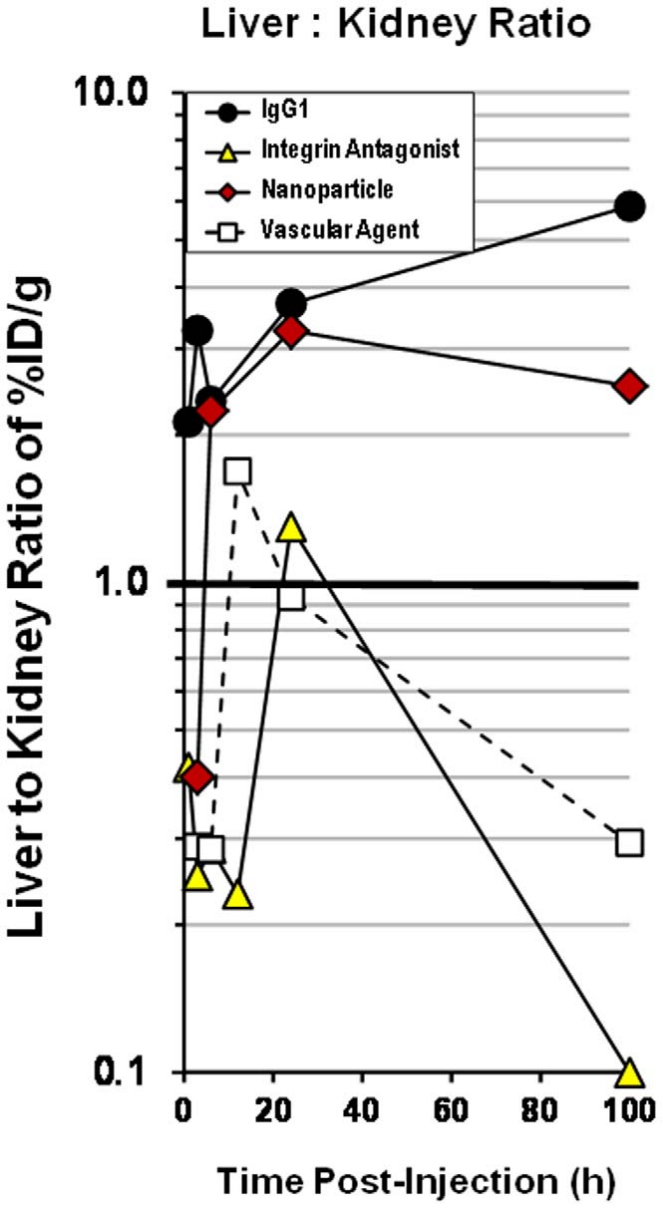
Determining kinetic changes in liver:kidney ratios. Mice from the study in Figs. 6 and 7 were further assessed for kinetic changes in the ratios of liver to kidney signal as a way of classifying each agent by its dominant route of tissue clearance. Plasma-corrected fluorescence signal in the liver and kidney (*i.e.* signal in the tissue only) was used to determine plasma PK corrected liver:kidney ratios.

Further analysis of liver:kidney ratios (Fig. 8) provided a relative understanding of the metabolic fate of each of the agents. Again, the corrected %ID/g values presented a picture of early kidney clearance of vascular, nanoparticle, and integrin antagonist agents. For the vascular and nanoparticle agents, this appeared to be due to the rapid clearance of fluorophore and agent metabolites at early time points and agreed with the kinetics of bladder signal in the animals. At later times, the vascular agent showed evidence of both liver and kidney roles in clearance. In the case of the integrin antagonist, the kidneys appeared to be the major route of clearance, accounting for the high bladder signal and relatively poor plasma pharmacokinetic profile. In contrast, the nanoparticle agent and IgG1 both showed a predominant liver signal, a pattern of distribution expected for these agents.

### Fluorescence volume assessment as an indicator of volume of distribution

**Figure 9:**
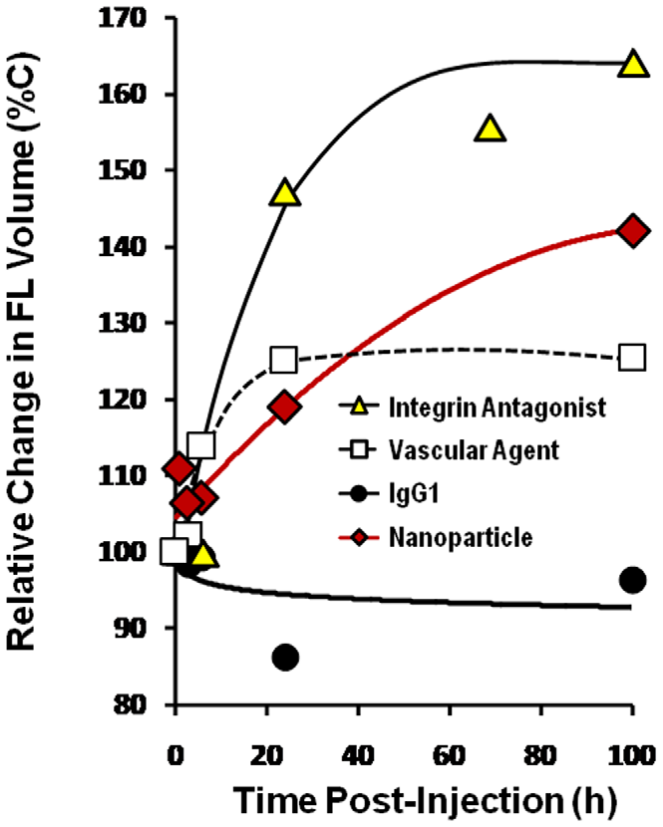
Determining relative fluorescence volume of distribution changes of different imaging agents. Fluorescence data from the study in Fig. 6 was further analyzed for kinetic changes in the volume of fluorescence at different concentrations within the body as determined by FMT2500. Fluorescence volume changes for four different concentration ranges were represented in graphical form as normalizations to starting volumes.

By standard pharmaceutical calculations, the volume of distribution (also known as the “apparent volume of distribution”), is the theoretical volume of fluid into which an administered drug would have to be diluted to produce the resulting plasma drug levels. Although volume of distribution has no absolute physiological meaning (*i.e.* does not provide actual physiologic volumes), it has been a useful pharmacokinetic parameter to predict the extent of distribution into bodily water or body fat. FMT quantification of fluorescence volume (mm^3^) in living mice would, in theory, provide a more direct surrogate for volume of distribution calculations, and results for four fluorescent agents are shown in Figure 9. As fluorescent agents passively extravasate into tissue, it would be expected that their concentrations would decrease, while their volumes would increase. At early time points, we expected an overestimation of fluorescent volumes due to the “partial volume effects” seen when analyzing fluorescent signal (*i.e.* the increase in *apparent* voxel numbers when signal occurs on the interface between multiple voxels in 3 dimensions). This effect would be expected to be particularly strong when applied to imaging tissue as fine and widespread as the vascular system, and it is interesting to note that all four agents showed approximately 7000–8000 mm^3^ of total fluorescence volume at time zero, the time at which all fluorescence would be presumed to be in the vasculature. Assuming that a 25 g mouse has approximately 2 mm^3^ of blood, this starting fluorescence volume corresponds to an approximate 4-fold overestimate of blood volume.

For IgG1, the volume of fluorescence changed little over time (∼0% increase), with the loss of plasma signal balanced by liver tissue accumulation (Fig. 9). The relatively high tissue signal in the absence of dramatic increases in fluorescence volume shows that IgG1 extravasates to some degree but poorly penetrates into tissue, as expected for immunoglobulin [24]. The nanoparticle fluorescence volume increased ∼40% with half of this volume due to very low concentration fluorescence. The vascular agent showed a somewhat lower volume profile compared to the nanoparticle, and 75% of the volume was due to the lowest levels of fluorescence. As expected because of its low molecular weight, the integrin antagonist showed the greatest change, increasing in fluorescence volume by 60% at 100 h, roughly. Adjusting for partial volume effects, approximations of 100 h fluorescent volumes can be made for the integrin, vascular, nanoparticle and IgG1 agents (8.0, 4.6, 6.2, and 2.0 mm^3^, respectively) and compared to approximate standard measures for vascular volume (∼2 mm^3^), total body water (∼18 mm^3^), and total body fat (∼4 mm^3^) in a 25 g mouse [25].

## Discussion

Non-invasive preclinical imaging techniques are quickly becoming important tools in biomedical research, leading to significant advances in the ability to monitor disease progression and the impact of therapeutic intervention in a variety of disease areas. With the recent advances in NIR tomographic imaging technology we have been able to demonstrate the capabilities of fluorescence molecular tomographic imaging to detect and quantify fluorescent biomarkers of disease in sites of cancer, inflammation, and infectious disease [16], [17], [26], [27], [28].

Recent studies by Nahrendorf et al. [30] have further advanced the level of validation of FMT imaging by performing hybrid PET-FMT imaging studies, using targeted agents labeled both with a NIR fluorophore and ^18^F or ^64^Cu. The seamless integration of tomographic FMT and PET datasets showed excellent spatial co-registration and quantitative correlation, supporting the utility of FMT imaging as a non-radioactive surrogate for PET imaging in preclinical studies. To further take advantage of the robustness of FMT imaging, and the use of fluorescence rather than radioactivity, the present studies support the expansion of the horizons of preclinical FMT imaging into quantitative biodistribution assessment. FMT imaging of fluorescent agents of vastly different characteristics, from small molecule to large proteins to nanoparticles, yielded quantitative time course information regarding differential kinetics, organ distributions, and clearance routes. By incorporating parallel blood pharmacokinetic data into the imaging analysis, we were further able to separately quantify the vascular signal and the extravasated tissue signal. This analysis, although not the standard for PET biodistribution studies (which does not remove the blood contribution), provided useful information regarding the rate of extravasation into tissues and was particularly helpful in understanding biodistribution patterns at very early time points at which agents remained high in circulation. Subtracting circulating fluorescence from tissue fluorescence was also critical in the proper interpretation of biodistribution results in some instances; without PK correction, skin, heart, and lung distribution were often grossly overestimated due to the relatively high contribution of the circulating signal to the overall imaging results. Additional techniques are being explored that may allow the selective analysis of vascular and tissue signal without the need for parallel pharmacokinetic studies, further streamlining the biodistribution imaging process. Other readouts, such as liver:kidney ratios and fluorescence volume of distribution will need extensive validation to establish their utility as robust readouts.

The major utility of FMT technology in biodistribution imaging is likely to be the application to the design and modification of antibodies or large protein therapeutics, as well as to the characterization of novel optical imaging agents of any size. Such an approach, in many instances, could be an option when rapid biodistribution screening is needed. Fluorescence-based tomographic imaging by FMT uses no radioactivity, minimally affects performance of large proteins, and presents an easy and user-friendly option with no need for special safety considerations or animal quarantine. Fluorescence approaches, however, may be less useful than PET or SPECT imaging in the study of very low molecular weight drug biodistribution, due to the significant impact on function, pharmacokinetics, and biodistribution when you double or triple a drug’s size by fluorophore conjugation.

Although disease site fluorescence has been approached carefully in many published studies, little work has been done to provide the full picture of agent accumulation in non-target tissues other than comparisons to specific control sites. With the current capabilities of FMT imaging, we are now examining agent targeting to sites of disease in the context of general biodistribution, helping in the overall understanding of both the disease biology and specificity of agent deposition. Further addition of kinetic readouts can be added to understand rates of agent deposition to specific sites (in the case of imaging time points post agent injection) and to track the progression of disease (when imaging at different times following initiation of disease). Multi-channel capability for FMT imaging also allows the simultaneous imaging of multiple agents, further decreasing the need for large cohorts of animals.

In conclusion, we have demonstrated the ability of the FMT imaging to non-invasively visualize and quantify whole-body tissue biodistribution in a robust and consistent manner. The consistency of the quantitative tomography, as well as its excellent correlation with *ex vivo* assessment of organ tissue signal, provides a powerful tool for quantifying the distribution and metabolic fate of FR- and NIR-labeled agents. FMT imaging in antibody and imaging agent research, utilizing NIR fluorescent labels, will provide a useful, non-invasive tool for understanding their distribution and metabolism.

## Materials and Methods

### Ethics statement

All experiments were performed in accordance with the recommendations in the Guide for the Care and Use of Laboratory Animals of the National Institutes of Health. The protocol (#01-0904) was approved by PerkinElmer’s (In Vivo Imaging Division) IACUC guidelines for animal care and use. No invasive or surgical procedures were used in these studies, but all imaging activities were performed under appropriate anesthesia to minimize animal distress.

### Experimental animals

For whole body biodistribution studies, specific pathogen-free female Nu/Nu mice (4–6 weeks of age) were obtained from Charles River (Wilmington, MA) and housed in a controlled environment (72°F; 12∶12-h light-dark cycle) under specific-pathogen free conditions with water and food provided *ad libitum*. For PK studies, specific pathogen-free female CD-1 mice (6–8 weeks of age) were obtained from the same vendor and kept under the same conditions as above.

### Fluorescent agents

Commercially available (PerkinElmer Inc., Boston, MA) near infrared imaging agents and custom conjugates were used to optimize and perform biodistribution studies (summarized in Table 1). All agents, except for GastroSense 750 were dosed at 2 nmol/mouse intravenously. GastroSense 750 (0.25 nmol/mouse PO), is a gastrointestinal imaging agent, AngioSense® 680 is a vascular imaging agent; ProSense® 680 detects regions of increased lysosomal cathepsin activity; ReninSense 680 FAST is activated by kidney renin; IntegriSense® 750 accumulates in regions of αvβ3 integrin upregulation; AngioSPARK® 750is a nanoparticle-based vascular imaging agent; VivoTag® 680XL (VT680XL) is a FR/NIR fluorophore.

**Table 1:** Characteristics of Fluorescent-Labeled Agents.

Agent	Description	Fluorophore per molecule	PeakEx/Em	Approximate Size	Imaging Utility
VivoTag-680XL	Fluorophore	1	668/688	1860 g/mol	Labeling of proteins
BSA-VT680XL	Bovine Serum Albumin	3	668/688	72,000 g/mol	Liver contrast agent
IgG1-VT680XL	Control IgG1	3	668/688	160,000 g/mol	Control for specific IgG1
ProSense 680	Cathepsin-Activatable	Multiple quenched	680/700	400,000 g/mol	Detection of disease-related proteases
ReninSense 680 FAST	Renin-Activatable	2 quenched	675/693	43,000 g/mol	Detection of kidney renin changes in hypertension
AngioSense 680	Vascular Agent	1	680/700	250,000 g/mol	Vascular leak in angiogenesis & edema
GastroSense 750	Oral Agent	1	750/770	40,000 g/mol	Gastric emptying, gastrointestinal contrast
IntegriSense 750	Integrin Antagonist	1	755/775	1280 g/mol	αvβ3 upregulation in disease
AngioSPARK 750	Nanoparticle	Multiple	750/775	20–50 nm	Vascularity, vascular leak

Custom protein-fluorophore conjugates were produced using bovine serum albumin (BSA) that was purchased from Sigma-Aldrich (ST. Louis, MO), and murine IgG1 purchased from Rockland (Gilbertsville, PA) to provide reference agents for biodistribution studies. N-hydroxysuccinimide (NHS)-ester labeling of proteins was performed using the 680 nm fluorophore (VivoTag 680XL, PerkinElmer). Briefly, the NHS ester moieties of VivoTag 680XL (pH 7 for 1 h at room temperature) reacted with primary amino groups of proteins to form stable amide bonds. In this manner, a small number of lysine residues (generally 1 to 5) in antibodies and other proteins can be labeled with fluorophore, with free fluorophore removed by size exclusion chromatography. Conjugation ratios were easily assessed by measuring absorbance of the conjugates at 280 and 680 nm with appropriate extinction coefficients to determine protein and fluorophore concentration, respectively. Labeling for both BSA (BSA-VT680XL) and IgG1 (IgG1-VT680XL) achieved ∼3 fluorophores per protein molecule, and these conjugates were dosed in animals at 2 nmoles of fluorophore/mouse IV. These conjugates showed excellent stability when stored at 4C and, upon *in vivo* injection, showed no evidence of release of free fluorophore.

### *In vivo* biodistribution imaging

Nu/Nu mice were injected IV with 2 nmoles of FR/NIR fluorescent agents and imaged at time 0, 1, 3, 6, and 24 h following injection. At the time of imaging, mice were anesthetized by inhalation in a chamber containing a mixture of isoflorane and oxygen then imaged using the FMT 2500 fluorescence molecular tomography *in vivo* imaging system (PerkinElmer Inc., Boston, MA). For whole body imaging, the anesthetized mice were placed in the supine position, centrally in the imaging cassette, to capture the whole body (excluding the head) of the animal within the imaging scan field of the imaging system. For imaging the head, the mice were placed in the prone position in the imaging cassette for a separate scan. After positioning the mouse, the imaging cassette was adjusted to the proper depth to gently restrain the mouse and then inserted into the heated docking system (regulated at ∼37°C) in the FMT imaging chamber. A FR/NIR laser diode transilluminated (*i.e.* passed light through the body of the animal to be collected on the opposite side) the animal body, with signal detection occurring via a thermoelectrically cooled CCD camera placed on the opposite side of the imaged animal. Appropriate optical filters allowed the collection of both fluorescence and excitation datasets, and the multiple source-detector fluorescence projections were normalized to the paired collection of laser excitation data. The entire image acquisition sequence took approximately ∼8–10 min (torso) and ∼2–3 min (head) per mouse.

### FMT reconstruction and analysis

The collected fluorescence data was reconstructed by FMT 2500 system software (TrueQuant, PerkinElmer Inc., Boston, MA) which compensates for the effects of tissue heterogeneity on light scattering [29], allowing for the quantification of fluorescence signal within multiple organ regions. Three-dimensional regions of interest (ROI) were drawn to encompass lung, heart, liver, stomach, kidney, intestine, and bladder tissue regions. For visualization and analysis purposes, FMT 2500 system software provided 3D images and tomographic slices. The total amount of fluorescence (in pmoles) in different organ sites was automatically calculated relative to internal standards generated with known concentrations of appropriate FR/NIR dyes. To prevent loss of low-level fluorescence following dissemination of agents into tissues, no thresholding of fluorescence datasets was applied. The percent injected dose (%ID) at each time point was determined relative to the initial imaging time point for each animal, assuming that combining whole torso and head scan at the start captured 100% of the pmols injected IV. In some studies in which only the full torso scan was collected, the starting quantification was defined as 85% ID (15% being the average contribution of head fluorescence in pmol using a variety of FR/NIR agents).

To determine the %ID/g in the different organs of living mice without the contribution from circulating blood, pharmacokinetic data was used to subtract blood levels from each organ at each time point. This was done by assuming that all organ signal at the first imaging time was due to fluorescence within the blood vessels of the organ as depicted in Figure 1. For agents in which significant bladder signal was detected at the first imaging time point (IntegriSense, AngioSense, and AngioSPARK), the %ID for bladder signal was used to adjust the starting %ID for the blood. For example, IntegriSense showed ∼10% of the injected dose in the bladder at the first imaging time, so blood levels were corrected to start at 90%. This was deemed an appropriate correction because bladder clearance will obviously affect blood levels. Minimal loss by excretion was seen for agents at this time. Time point by time point subtraction of the average PK curve (normalized to the first imaging time) from the whole organ tissue distribution curve effectively “zeroes” the organs at the start – a time when there has not been significant extravasation into tissue - and reveals the kinetics of extravasation into the organ tissue. Standard organ weights were determined in previous studies using mice of the same strain and size (brain 0.42 g, lungs 0.23 g, liver 1.0 g, intestines 1.22 g, heart 0.12 g, bladder 0.12 g, stomach 0.18 g, kidneys 0.15 g each). Tissue weight values per ROI were adjusted for liver and lung regions to account for only partial capture by the placed ROIs (liver 0.75 g, lungs 0.2 g).

### *Ex vivo* tissue assessment of biodistribution

After animals were imaged *in vivo,* they were sacrificed by carbon dioxide asphyxiation. The organs (brain, heart, lungs, liver, spleen, kidneys, stomach, intestines, skin, blood, and bladder) were removed post-mortem then imaged by fluorescence reflectance imaging using FMT 2500’s planar imaging capability and frozen at −80 C. For some animals, organs were also subjected to a homogenization procedure. The organ tissues were thawed in the dark, and then weighed. The appropriate volume of PBS (0.5 mL/mg of tissue) was added to each tissue prior to homogenization to an even consistency. Planar fluorescence reflectance imaging was used to measure the fluorescence from serial dilutions of each homogenate (10 uL/sample) to assure quantification was performed within ranges of fluorophore concentration not subject to intermolecular fluorescence quenching. Appropriate dilutions of normal tissue homogenates were used to measure background fluorescence which was subtracted from the final calculations.

### Pharmacokinetics

CD-1 or nude mice were injected IV with a known amount of fluorogenic agent. Terminal blood samples were collected by cardiac puncture from each mouse at the appropriate time point following carbon dioxide asphyxiation. A minimum of two mice were sampled for each time point. Plasma was collected by centrifugation and diluted 1∶2 in DMSO to assure capture of maximal fluorescent signal. Plasma fluorescence was measured using a fluorescence plate reader (Molecular Devices, Sunnyvale, CA). The data was normalized to a standard curved prepared with known concentrations of the agent to quantify blood levels in ug/ml, and results were converted to percent of injected dose. Mouse inter-strain differences in pharmacokinetic profiles were well within the range of variability seen within mouse strains.
